# Scenario Analysis and Path Selection of Low-Carbon Transformation in China Based on a Modified IPAT Model

**DOI:** 10.1371/journal.pone.0077699

**Published:** 2013-10-29

**Authors:** Liang Chen, Zhifeng Yang, Bin Chen

**Affiliations:** State Key Joint Laboratory of Environmental Simulation and Pollution Control, School of Environment, Beijing Normal University, Beijing, P R China; DOE Pacific Northwest National Laboratory, United States of America

## Abstract

This paper presents a forecast and analysis of population, economic development, energy consumption and CO_2_ emissions variation in China in the short- and long-term steps before 2020 with 2007 as the base year. The widely applied IPAT model, which is the basis for calculations, projections, and scenarios of greenhouse gases (GHGs) reformulated as the Kaya equation, is extended to analyze and predict the relations between human activities and the environment. Four scenarios of CO_2_ emissions are used including business as usual (BAU), energy efficiency improvement scenario (EEI), low carbon scenario (LC) and enhanced low carbon scenario (ELC). The results show that carbon intensity will be reduced by 40–45% as scheduled and economic growth rate will be 6% in China under LC scenario by 2020. The LC scenario, as the most appropriate and the most feasible scheme for China’s low-carbon development in the future, can maximize the harmonious development of economy, society, energy and environmental systems. Assuming China's development follows the LC scenario, the paper further gives four paths of low-carbon transformation in China: technological innovation, industrial structure optimization, energy structure optimization and policy guidance.

## Introduction

As mutual challenges for human society, the climate warming caused by human activities and possible catastrophic results have been highly recognized all over the world. Reduced greenhouse gas emissions for low-carbon development may be the new standard for all countries and may restrict relevant economic and political activities. It is quite notable that almost every country has begun to study future greenhouse gas emission and mitigation options with greater zeal in preparation for approaching international negotiations. This has especially been the case in China. As the largest developing country, China's energy consumption and CO_2_ emissions increased sharply along with the rapid economic growth over the years [1]–[3]. From 1990 and 2010 China’s total energy consumption has increased 5.7% annually, and CO_2_ emissions grew by nearly 2.5 times [4]. In 2007, the total CO_2_ emission from fossil fuel consumption in China exceeded that of the United States, which made China the world's largest CO_2_ emitter [5], [6].

In response to the increasing pressure for CO_2_ emissions reduction, at the Copenhagen Climate Change Conference in 2009 China put forward a climate change mitigation target of 40%–45% reduction of CO_2_ emission intensity by 2020, increasing the share of non-fossil fuels in primary energy consumption to around 15% by 2020 compared with the 2005 level [7]. Moreover, the Chinese government published “China’s Pathway towards a Low Carbon Economy for 2050” where technology and other factors will play a significant role in the reduction of carbon emissions [8] that needs to be taken into account. These emission reduction targets and low-carbon paths indicate that “CO_2_ emission reduction” as a restrictive index for China’s social development has been incorporated into assessment systems and become an important factor for China in the strategy of sustainable development. Therefore, forecasting the influences of low-carbon policies and measures and systematically analyzing the feasibility of China’s carbon emission target in different development stages under different scenarios are significant decision-making references for establishing scientifically sound scenarios of CO_2_ emissions in China, as to guide and promote carbon emission targets and explore optimized paths of low-carbon development.

The “scenario” refers to the description and forecast of future situations and of situations developed from original state to future state. Common forecasting methods (such as time series analysis, multiple objective linear programming, genetic algorithms, neural network models and chaotic dynamics models) mainly focus on the influence from quantitative factors, but it is impossible to evaluate the factors that cannot be quantified, e.g. policy orientation in a scientific mode. However, scenario analysis can effectively avoid the limitations of traditional analysis methods. Assuming that a certain phenomenon or trend may continue into the future, and the possible situation or relevant consequence is evaluated. It is significant not because the future state of objects of study is accurately forecasted but the possible states in different trends can be investigated, compared and studied. Thus, deep analysis can be realized by the comparison of scenario settings so as to furnish scientific proposals and decision-making references for the path selection of future development [9]–[11].

The IPAT accounting model, first proposed in the early 1970 s [12], [13], is a widely applied model to analyze and predict the relations between human activities and the environment. The IPAT model can not only finish the quantitative analysis of environmental impacts from population, economy and technology, but also simulate the development of population, economy and other aspects [14], [15], with the advantages of easy understanding and wide utilization for analyzing key driving forces of anthropogenic environmental change [16]–[19]. The model classifies all factors that have effects on the environment into three drivers: Population growth (*P*), Affluence (*A*) represented by consumption or production patterns, and Technology (*T*) as environmental impacts per unit of consumption or production to capture the effects of all other factors. Each is assumed to have proportional effects on the environment. IPCC [20] discussed the IPAT model and its application to future emissions scenarios covering a wide range of the main driving forces from demographic to technological and economic developments. Many scholars have gathered a substantial amount of literature on scenario analysis and index decomposition analysis, upon which the IPAT model is based, for energy use and environmental emissions [21]–[23].

However, the proportional assumption between factors and environmental indicators cannot be tested by the IPAT model itself [24], [25]. For example, technology (*T*) is generally derived from the environmental impacts divided by the other two drivers: population growth (*P*) and affluence (*A*). In recent years, recognizing this limitation, much research has been done to modify the IPAT framework by disaggregating one driver into more factors in the equation [26]–[28]. Since then, IPAT has had more widespread application in analyzing and predicting future primary energy demand and CO_2_ emissions [19], [29]–[31], giving insight to policy makers in creating feasible and practical climate polices. This study aims to forecast the influences of low-carbon policies and systematically analyze the feasibility of China’s carbon emission target in different development stages and under different scenarios for exploring the optimized path of low-carbon development in China.

## Methodology

The IPAT model was reformulated as the “Kaya identity” [32], which is the basis for calculations, projections, and scenarios of greenhouse gases (GHGs) implemented by the Intergovernmental Panel on Climate Change (IPCC) in 1996 [33]. The basic equation of IPAT model can be expressed as follows:
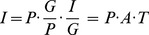
(1)


In equation (1), *I* indicates environmental load; *P* indicates population; *G* indicates total GDP; *A* indicates GDP per capita, namely, richness degree; *T* indicates environmental load per unit of GDP, namely, technology level.

According to the object of study herein, the basic model is extended and decomposed, *C* is made to stand for *I* to indicates environmental impact due to energy consumption, namely, CO_2_ emissions as the result of terminal energy consumption; *c* is used to stand for *T* to indicate CO_2_ emissions per unit of GDP, namely, carbon emission intensity. Therefore, Equation (1) can be expressed as below:
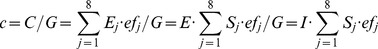
(2)

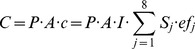
(3)Where index *i* = 1, 2,…, 8 respectively denote 8 types of fossil fuels. The meanings of other variables in Eq. (2) and Eq. (3) are described in Table 1.

**Table 1:** Definitions of each variable in Eq. (1–3).

Variable	Definition	Variable	Definition
*C*	Total amount of CO_2_ emissions (ton)	*P*	Total population
*A*	GDP per capita (yuan)	*G*	GDP (yuan)
*E*	Total amount of fossil fuel consumed (tce)	*c*	*c* = *C/G*, CO_2_ emission intensity (*tC/yuan*)
*E_j_*	The amount of fossil fuel *j* consumed (tce)	*I*	*I* = *E*/*G*, energy intensity (*tce/yuan*)
*ef_j_*	carbon emissions coefficient for fossil fuels *j* (tC/tce)	*S_j_*	*S_j = _ E_j_*/*E,* energy structure

Seen from Equation (3), CO_2_ emissions vary due to the influence from population (*P*), GDP per capita (*A*), energy structure (*S*) and energy intensity (*I*), namely, energy technology (*ef_j_* indicates carbon emissions coefficients of different energy resources, generally existing in the form of a constant). These four factors have significant influences on China’s CO_2_ emissions and are selected as scenario analysis indexes in this study to structure the extended model for the scenario analysis. If *C_0_* indicates energy-related CO_2_ emissions in the base year, the annual growth rate of GDP per capita is *m*, the natural growth rate of population is *r*, the rate of technology progress in energy is *k*, 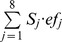 is defined as optimization coefficient of energy structure and its optimization rate is *f*, CO_2_ emissions in target year (*T*) will be:
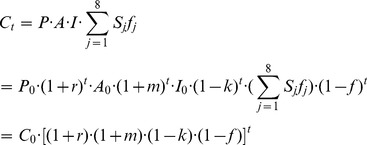
(4)


In case of 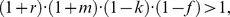 this situation indicates that the rapid growth of population and GDP offsets the emission reduction due to technology progress in energy and energy structure optimization, and that CO_2_ emission rises under the combined action of four factors if compared with that in base year.

In case of 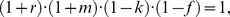 this situation indicates that the emissions reduction due to energy technology progress and energy structure optimization offsets the energy consumption promoted by population growth and GDP growth. That means the CO_2_ emissions achieve zero growth due to the action of four factors above.

In case of 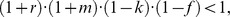 this situation indicates that the degree of energy conservation and emissions reduction due to energy technology progress and energy structure optimization exceeds that of the energy consumption and emissions increase promoted by population and GDP growth. CO_2_ emissions and energy consumption will thus be gradually reduced with the action of population and GDP growth.

## Scenario and Parameter Design

### Main Premises and Sources for Scenario Design

Scenario analysis is widely used by many scholars domestically and abroad to discuss indicators’ trends, which are easily measured by statistical data and convenient for use in policy adjustment. The rationale and principle of scenario assumptions are based on the future scenarios of economic and social development objectives. The descriptions of the scenarios which follow are in terms of development plans in society, economy, energy and environment, although many of them will need substantial development from their present status. Finally, the scenarios could be explained in terms of a number of different broad social and cultural drivers, or states of the economic background. Therefore, the scenario assumption is some level of scientific forecast of possible phenomenon or development trend in the future, according to the current situation and future objectives.

This section gives the following ideas of scenario assumptions learned from some relevant literature [9], [34]–[38] to analyze energy demand and CO_2_ emissions before 2020. The future scenario frameworks of China's socio-economic factors are firstly described, and possible policy drivers of this development mode and the behavior changes implied by the scenario are further discussed in each scenario. Then, the parameters of the main scenario indicators are set according to the influencing factors decomposed by IPAT extended model.

The Chinese government has reconsidered and adjusted its development policies every fifth year since 1953, called the *Five-Year Plan*, to control its socio-economic development. The time span of scenario assumption in this study ranges from 2007 to 2020, which covers the period of China’s 12^th^ *Five-Year Plan* (2011–2015). With 2007 as the base year, the stages of scenario prediction are divided into the short-term step (2011–2015) and long-term step (2016–2020) in accordance with this *Five-Year Plan.* The strategic and development targets in the 12^th^ *Five-Year Plan* are considered to be the key reference for scenario assumptions and parameters design. First of all, the following important premises and background from the plan are assumed to be shared among all scenarios:

1. There has been no sign in recent years that the Chinese government will significantly change its population policy. As a result, China’s population growth is assumed to follow past trends.
2. China’s CO_2_ emissions have greatly increased in recent years. From 2001 to 2008, its average annual rate of increase was 11.8%–much greater than that of the rest of the world (which during the same period was 3.37%). The share of China’s CO_2_ emissions in the world total also increased from 12.45% in 2001 to 21.55% in 2008. China has become the largest CO_2_ emitter in the world since 2007 [4]–[6]. The country is going through more and more emission pressures both domestically and from overseas.
3. Since the reform and expansion policy in late 1978, the national economy has developed with remarkable speed. However, this supernormal development was achieved at the cost of environment destruction, not only because some problems existed in the environmental management system but also because China paid primary attention to economic gain at the present stage [39].
4. The period of 2011–2015 is that covered by China’s 12^th^ *Five-Year Plan*. In accordance with the strategic plans of Chinese government, the coordinated development of the environment and economy will be one of its most important goals. The Chinese government has decided to control the growth of CO_2_ emissions and to improve energy efficiency. More attention will be directed from economic growth to environmental protection.
5. In order to quantify its development strategic plans and conveniently compare them to other periods, the Chinese government has designed its development targets by setting some key development indicators (e.g. growth rate of GDP). Following this idea, the change rates of *I*, *P*, *A*, and *T* considered as the starting points of the scenario design should scientifically refer to relevant quantitative targets of government.

### Scenario Design

Based on these facts, we designed four scenarios of CO_2_ emissions for the period before 2020 in this study. Each scenario represents a different path, which is possible in China’s future development based on various policies and measures.

#### Scenario 1

The first scenario (Business as usual scenario,BAU), in which the measure to deal with climate changes is not taken, is a future scenario designed for possible development modes. The economic growth is the uppermost driver of carbon emission, and industrialization is urged without considering the energy consumption structural optimization. The style that emphasizes development while neglecting environment remains unchanged. According to former scenario descriptions and future development plans in China, economic growth is taken as the major target for social development and the annual GDP growth rate is assumed to 9.8% during 2010–2015, and 8.5% during 2016–2020. Considering China’s constant population policy, the population growth is assumed to have a growth rate of 0.60% during 2010–2015, and 0.53% during 2016–2020. The technical innovation caused by industrialization improves to some extent, maintaining the stable progress rate of 2%, and the energy efficiency advances in accordance with original structure optimization rate of 0.9%.

#### Scenario 2

The second scenario is an energy conservation scenario (Energy efficiency improvement scenario,EEI) in which current energy conservation and emission reduction have been considered, but a countermeasure for CO_2_ emission variation is not explicitly carried out. Compared with BAU scenario, the EEI scenario has been concerned with the change of economic growth mode and taken special measures for energy conservation and CO_2_ emission reduction to continue present energy policy. However, some complex contradictions among energy, environment and economy are still inevitable. According to the former scenario description, the economic growth mode has not been radically changed but with a certain degree of slowing down, and the GDP growth rate is assumed to drop to 8.2% during 2010–2015, and 7.5% during 2016–2020. The technical innovation capability is continuously promoted by the energy efficiency policy so that the technology progress rate significantly increases over 3% during 2016–2020. Other scenario parameters are assumed to not differ considerably from BAU.

#### Scenario 3

The third scenario is low carbon emission scenario, also called low carbon scenario for short (Low carbon scenario, LC), based on the integration of energy safety, sustainable development and low carbon measures, etc. China’s social, economic and environmental development demands in the future are fully considered, and low-energy consumption and low-carbon emissions are realized according to actual situations in China. Under the LC scenario, the economic development mode is significantly changed, and economic growth has not been set as the primary target. The GDP growth rate is assumed to further decline below 7% during 2016–2020. The low-carbon transformation in technology leads to a continuous increase of progress rate: to 3.8% during 2010–2015, and 8.5% during 2016–2020. Energy efficiency is similar to that of the baseline scenario with the tiny decline in structure optimization rate. Overall the emission targets of reduction by at least 45% of CO_2_ emission intensity compared to 2005 will be achieved.

#### Scenario 4

Similar to LC Scenario, the fourth scenario is enhanced low carbon scenario (ELC) under the background that all countries make mutual efforts to mitigate climate change. Under ELC scenario, the government increases attention and investment to better boost low-carbon transformation of economic development mode, and the tertiary industry gradually dominates the economic structure. The GDP per capita will be expected to reach 10000 dollars in 2020. Technical innovation capability is also further enhanced, and new energies’ technology achieves a breakthrough. The share of non-fossil energy will be more than 15%. Clean energy development and substitution as well as carbon capture and storage (CCS) are further widely applied, with energy efficiency up to the world average level. China's CO_2_ emissions intensity will be decreased by 60% in 2020.

All the scenarios will be based on previous development targets and projected policy direction, and will significantly differ in their economic model, technical compositions and energy efficiency. This could result in changes in energy demand and consumption, economic output, and CO_2_ emission. By comparing these scenarios, the energy-saving and CO_2_ abatement potential by different development paths can be acquired.

### Parameter Definition

Seen from the decomposition results of the IPAT model (Eq.3), population (*P*), GDP per capita (*A*), energy structure (*S*) and energy technology (*I*) have significant influences on China’s CO_2_ emissions and are selected as main scenario indicators of future scenario framework. The definitions of corresponding parameters for each indicator are given with full consideration of the common premises and previous scenario design (Table 2).

**Table 2:** Scenario description and parameter definition.

Scenarios	Policy and measures	Scenario description	Steps	Parameters a, b			
				*m*	*r*	*k*	*f*
Scenario 1: Business asusual scenario (BAU)	It remains the original tendency, which does nottake the measures to deal with climate changes.Economic growth is taken as the uppermost targetfor social development.	The economic growth is the major driver of carbon emission, andindustrialization is constrained without considering the energyconsumption structural optimization. The technical innovation andenergy efficiency improve to some extent maintaining a stableprogress rate.	Short-term(2011–2015)	9.80	0.60	1.98	0.90
			Long-term(2016–2020)	8.50	0.53	2.11	0.89
Scenario 2: Energyefficiency improvementscenario (EEI)	The policy and countermeasure for low-carbontransformation is not particularly taken, but takingsome special measures for energy conservation andCO_2_ emission reduction to continue presentenergy policy.	The economic growth mode has not been radically changed butwith a certain degree of slowing down. The technical innovationcapability is continuously promoted by the energy efficiency policywith significant increase of progress rate. Other scenario parametersare assumed to not been particularly changedfrom baseline scenario.	Short-term(2011–2015)	8.20	0.60	2.78	0.89
			Long-term(2016–2020)	7.50	0.53	3.56	0.87
Scenario 3: Low carbonscenario (LC)	The integration of energy conservation, low-carbontransformation and sustainable developmentmeasures are fully considered. The relevant policyand countermeasure are carried out reasonablyaccording to actual situations in China.	The economic development mode is significantly changed, leadingto the further decline of GDP growth rate. Continuous increase oftechnology progress rate is boosted by the attention of low-carbondevelopment. Energy efficiency is similar to that of the baselinescenario with the tiny decline. The targets of reduction by at least45% of CO_2_ emission intensity compared to 2005 will be achieved.	Short-term(2011–2015)	7.42	0.60	3.83	0.84
			Long-term(2016–2020)	6.86	0.53	4.65	0.82
Scenario 4: Enhanced lowcarbon scenario (ELC)	All countries make mutual efforts to mitigate climatechange, under the background of which Chinesegovernment increases attention and investmentto better boost low-carbon transformation.	The tertiary industry gradually dominates the economic structure.New energies’ technology achieves a breakthrough, and the share ofnon-fossil energy will be more than 15%. Clean energy developmentand substitute as well as carbon capture and storage (CCS) arefurther widely applied. China's CO_2_ emissions intensity will bedecreased by 60% in 2020.	Short-term(2011–2015)	6.50	0.60	5.24	0.78
			Long-term(2016–2020)	5.47	0.53	5.56	0.75

a Parameters definition: *m,* growth rate of GDP per capita (%); *r*, population growth rate (%); *k*, technology progress rate (%); *f*, energy structure optimization rate (%).

b Data sources: The values of parameters are calculated or assumed based on the references from CCAP [44], [45], CAS [46], SCPRC [47], [48].

The policies and strategic plans, specifically the measures and objectives by these policies in scenario description, are the main basis for our assumptions. Related studies have given us abundant inspiration and information for scientific scenario and parameters design [36]–[38], [40]–[43]. The values of parameters for scenario indicators, including economical growth rate (*m*), population growth rate (*r*), technology progress rate (*k*) and energy structure optimization rate (*f*), are calculated or assumed based on the original data from both the policy research [44]–[46] by professional institutions and important development strategies such as China’s *Five Year Plan* by the government [47], [48].

## Scenario Analysis and Discussion

As seen in Table 3, China’s total energy consumptions (*E*), CO_2_ emissions (*C*) and other indexes differ significantly in different stages before 2020 under each scenario, for which the major cause is that dominant driving factors differ under different scenarios (Table 3). In terms of BAU, economic development is the dominant factor to promote the rapid growth of energy-related CO_2_ emissions so that its CO_2_ emission is 1.6 times of that under LC scenario and over 3 times of that under ELC scenario in 2020; regarding EEI scenario, the energy intensity is obviously lessened and total energy consumptions and CO_2_ emissions are largely reduced in different stages as compared with those under BAU scenario. By 2020, the total energy consumption will be reduced by 1.569×10^8^ tons of standard coal equivalent (tce), CO_2_ emissions will be totally reduced by 3.393×10^8^ tons and energy intensity will be 0.669 *tce/10^4^ yuan* (approximately equal to 0.41 tce/$); for LC scenario, through low-carbon development in technology, structure, consumption mode and other respects, the low carbon countermeasure for climate change is taken so that CO_2_ emission is clearly reduced. By 2020, CO_2_ emission will be reduced by 2.096×10^8^ tons in the EEI scenario and the carbon emission intensity is decreased by 46.36% on the level of base year. In the event that enhanced low carbon measure (ELC) is taken, the emission reduction target will be exceeded, but effective countermeasures for climate change will doubtlessly affect the rapid economic and social development in China. Hence, before 2020, the Chinese government shall strive to take various measures to reduce CO_2_ emissions, optimize industrial structure, gradually improve the contribution rate of low carbon technology progress, enhance industrial structure adjustment and the driving effect of technical factors, and reach the target of low carbon scenario.

**Table 3:** Prediction result of scenario indicators (2007–2020).

Scenario	Indexes^c^	Baseline	Short-term step					Long-term step				
		2007	2011	2012	2013	2014	2015	2016	2017	2018	2019	2020
**BAU**	*P*	13.25	13.58	13.66	13.74	13.83	13.91	13.98	14.06	14.13	14.20	14.28
	*G*	24.66	36.91	40.77	45.04	49.75	54.95	59.94	65.37	71.30	77.77	84.82
	*A*	1.860	2.718	2.985	3.277	3.598	3.951	4.287	4.651	5.047	5.475	5.941
	*E*	24.63	34.21	37.04	40.10	43.42	47.01	50.19	53.59	57.22	61.09	65.23
	*I*	0.999	0.927	0.908	0.890	0.873	0.855	0.837	0.820	0.802	0.786	0.769
	*C*	60.27	80.69	86.58	92.90	99.69	106.96	113.19	119.77	126.74	134.12	141.92
	*c*	2.444	2.186	2.124	2.063	2.004	1.946	1.888	1.832	1.778	1.725	1.673
**EEI**	*P*	13.25	13.58	13.66	13.74	13.83	13.91	13.98	14.06	14.13	14.20	14.28
	*G*	24.66	35.93	39.09	42.53	46.27	50.34	54.39	58.76	63.48	68.58	74.09
	*A*	1.860	2.650	2.867	3.102	3.356	3.631	3.904	4.197	4.511	4.850	5.213
	*E*	24.63	32.24	34.10	36.07	38.15	40.35	42.04	43.80	45.64	47.55	49.54
	*I*	0.999	0.897	0.872	0.848	0.824	0.801	0.773	0.745	0.719	0.693	0.669
	*C*	60.27	76.08	79.76	83.61	87.65	91.88	94.90	98.02	101.23	104.56	107.99
	*c*	2.444	2.117	2.040	1.966	1.894	1.825	1.745	1.668	1.595	1.525	1.457
**LC**	*P*	13.25	13.58	13.66	13.74	13.83	13.91	13.98	14.06	14.13	14.20	14.28
	*G*	24.66	34.24	36.97	39.92	43.11	46.55	49.97	53.65	57.60	61.84	66.40
	*A*	1.860	2.525	2.712	2.913	3.129	3.362	3.592	3.839	4.102	4.383	4.684
	*E*	24.63	30.35	31.52	32.73	33.98	35.29	36.13	36.98	37.86	38.76	39.68
	*I*	0.999	0.886	0.852	0.820	0.788	0.758	0.723	0.689	0.657	0.627	0.598
	*C*	60.27	71.75	73.88	76.08	78.34	80.66	81.90	83.15	84.42	85.72	87.03
	*c*	2.444	2.095	1.998	1.906	1.817	1.733	1.639	1.550	1.466	1.386	1.311
**ELC**	*P*	13.25	13.58	13.66	13.74	13.83	13.91	13.98	14.06	14.13	14.20	14.28
	*G*	24.66	32.87	35.19	37.67	40.32	43.16	45.74	48.47	51.37	54.44	57.69
	*A*	1.860	2.428	2.586	2.754	2.933	3.124	3.294	3.475	3.665	3.865	4.077
	*E*	24.63	27.49	27.88	28.28	28.69	29.10	29.12	29.15	29.17	29.20	29.22
	*I*	0.999	0.836	0.792	0.751	0.712	0.674	0.637	0.601	0.568	0.536	0.507
	*C*	60.27	65.07	65.48	65.90	66.33	66.75	66.31	65.87	65.43	64.99	64.56
	*c*	2.444	1.979	1.861	1.750	1.645	1.547	1.450	1.359	1.274	1.194	1.119

c Indexes definition: *P*, Population (*10^8^ persons*); *G*, GDP (*10^12^ yuan*); *A*, GDP per capita (*10^4^ yuan*); *E*, Total energy consumption (10^8^ tce); *I*, Energy intensity (*tce/10^4^yuan*); *C*, CO_2_ emissions (*10^8^ tons*); *c*, CO_2_ emission intensity (*tC/10^4^yuan*).

### Scenario Analysis of GDP

Since economic development is undergoing continuous and rapid growth in China, the baseline scenario assumes that China's current high growth will continue. Based on the target of the new three-step strategy, that is, GDP quadrupled in 2020 on the level of 2000, proposed by Chinese government, this paper lists the set target of quadrupling for the front three scenarios. Nowadays, China is in the rapid industrialization process. In accordance with economic development laws of developed countries, the economic growth will remain at a high speed through 2020. It is anticipated that China’s industrial structure will be gradually upgraded and the competitiveness of the energy industry and strategic emerging industries will be increasingly improved in future decades, but China’s economic growth can be still over 5% in continual adjustment on the whole, but will differ under different scenarios due to the influences of energy conservation and emission reduction policies.

**Figure 1:**
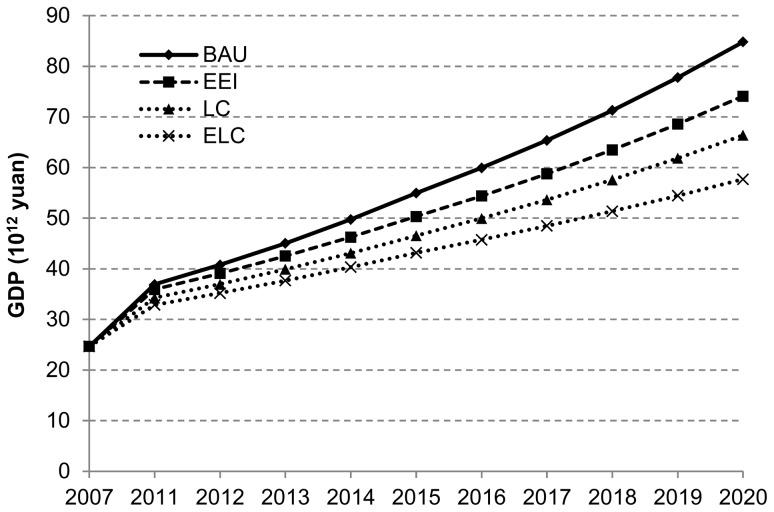
China’s GDP growth under BAU, EEI, LC and ELC scenarios (Scenarios are defined in Table 2).

As seen in Figure 1, GDP surges under the baseline scenario and will reach 84.82×10^12^ *yuan* which is respectively 10.73×10^12^ *yuan*, 18.43×10^12^ *yuan* and 27.13×10^12^ *yuan* more than GDP under EEI scenario, LC scenario and ELC scenario, and 1.28 times of GDP under LC scenario. GDP also increases at a high speed under EEI scenario, but the growth rate shall be less than the baseline scenario. Therefore, China’s carbon emission reduction measures in the future will largely restrict and affect the economic growth.

### Scenario Analysis of Energy Consumption and Energy Intensity

China’s total energy consumption will be in continuous growth in future decades, and rapid economic growth will inevitably stimulate a large energy demand so as to promote the growth of total energy consumptions. However, the growth rate of total energy consumptions will be restricted to some extent under EEI scenario, LC scenario and ELC scenario, due to energy conservation and emission reduction policy as well as low carbon technology.

**Figure 2:**
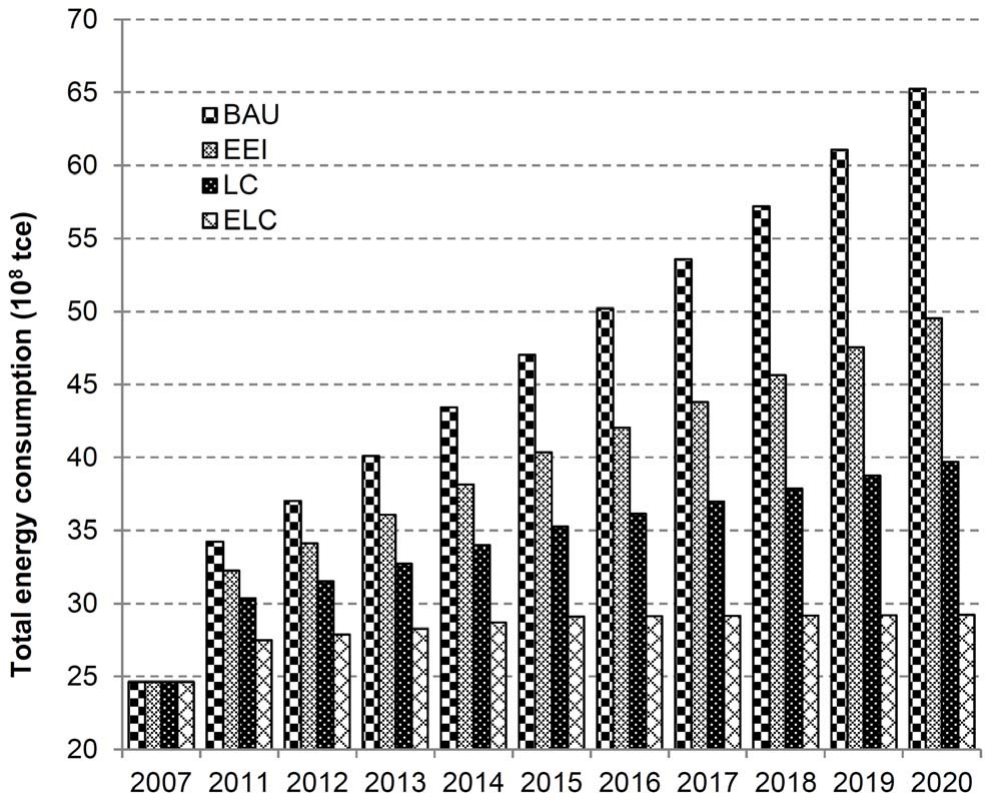
China’s total energy consumption under BAU, EEI, LC and ELC scenarios. (Scenarios are defined in Table 2).

The forecast indicates that total energy consumption will rise under four scenarios as a whole, but total energy consumption under LC scenario and ELC scenario fluctuated largely and relevant growth rate declined since advanced low carbon technology and energy conservation policy suppress economic growth and the demand for energy consumption is hugely restricted (Figure 2). When the technology is advanced to certain degree, total energy consumption will grow continuously and gradually on the basis of economic development. Furthermore, with the arrival of low-carbon era, the resource shortage will certainly result in a gradual reduction of fossil fuel consumption, with alternative energies such as water, wind, and biomass and renewable energy holding an increasingly larger share, thus, the optimization of energy consumption structure also affects China’s energy consumption variation in the future.

**Figure 3:**
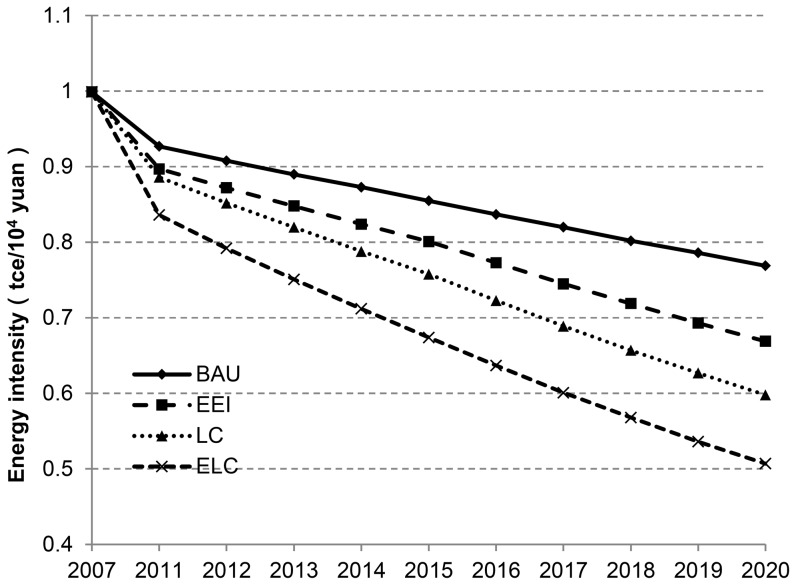
China’s energy intensity under BAU, EEI, LC and ELC scenarios. (Scenarios are defined in Table 2).

The scenario forecast of energy intensity will rely on the strength of energy conservation and emission reduction policy as well as the contribution value of technological progress under different scenarios. Hence, the energy intensity under the four scenarios will differ due to different impacts of the measures for energy conservation and emission reduction. Under the BAU scenario, although measures for energy conservation or emission reduction are not taken, with increasing improvement of technology levels, the energy intensity is also steadily reduced, though with a falling range far less than that of any other scenario. Therein, the energy intensity will be 0.589 *tce/10^4^ yuan* in 2020 under LC scenario: 0.171 *tce/10^4^ yuan* less than that under BAU scenario (Table 3, Figure 3). Therefore, positive carbon emission reduction measures and low carbon technology in the application will bring a better effect of energy conservation and emission reduction. The forecast reflects that the reduction of energy intensity under the four scenarios before 2010 was higher than that after 2010, but the drop of energy intensity under the four scenarios was steady and hardly fluctuated on a whole, for the technology brought an obvious effect of consumption and emission reduction in the initial stage when energy conservation and emission reduction policy was implemented; but after the initial stage, the policy orientation and technology progress result in a dropping energy intensity which would be gradually steady.

Since China is in the rapid industrialization process, low carbon technology progress plays a vital role in energy conservation, climate change mitigation and targeted carbon emission reduction in China. Thus, increasing technological expenditures, actively conducting international cooperation and promoting technology progress and innovation can effectively improve energy utilization ratio and reduce CO_2_ emissions. Such countermeasures and behaviors as energy conservation and emission reduction, and development of renewable energy have been implemented in China since “the 11^th^ five-year plan.” Besides, relevant carbon emission reduction technology and equipments, e.g. advanced clean coal technology, carbon capture and storage technology, solar use technology, biomass power generation & gasification technology and wind power generation technology are also positively developed and adopted in different industrial sectors so that low carbon technology is effectively improved and energy consumption per unit of GDP is clearly reduced. Compared with those in developed countries, the carbon emission reduction technology is weak and low carbon technology innovation is lagging in China nowadays, therefore, structuring low carbon technology systems and improving low carbon technology innovation are pivotal for China’s low-carbon development in the future.

### Scenario Analysis of CO_2_ Emissions

**Figure 4:**
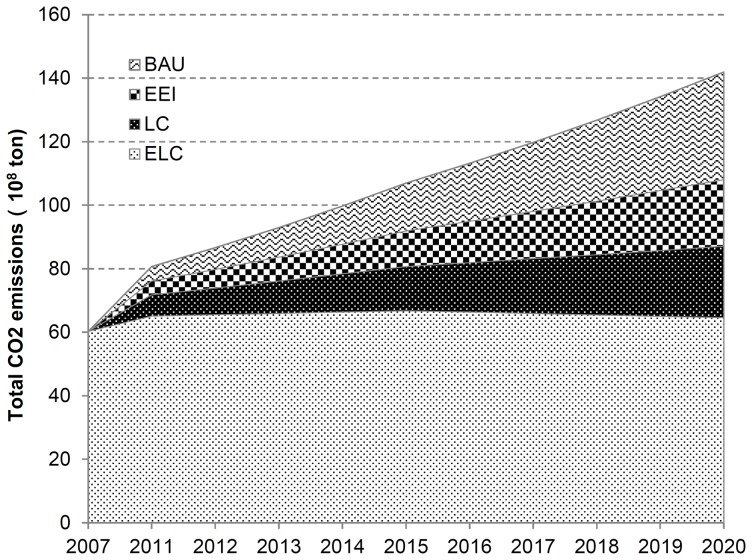
China’s energy-related CO_2_ emissions under BAU, EEI, LC and ELC scenarios. (Scenarios are defined in Table 2).

Under BAU scenario, CO_2_ emission maintains substantial growth and will be about 14.2 billion tons by 2020, and the growth rate is far more than that of any other scenario. In respect to the LC scenario, due to hampered carbon emission reduction technology and low investment in the initial stage when energy conservation and emission reduction policy is implemented, the CO_2_ emission underwent a significant growth before 2010. However, with continuous progress of carbon emission reduction technology, the whole growth trend of CO_2_ emission will clearly slow down in the long-term step. By 2020, CO_2_ emissions will be 8.703 billion tons under LC scenario–5.497 billion tons less than under the BAU scenario (Table 3, Figure 4). As regards to the ELC scenario, CO_2_ emissions first rise and then drop in development. With powerful climate change measures in implementation, the growth degree of CO_2_ emissions will shrink gradually and it is predicted that CO_2_ emissions will experience negative growth after 2015. Besides, economic growth apparently slows down, CO_2_ emission is decoupled from economic growth and as such, the ELC scenario may be realized with huge economic benefits.

The carbon emission intensity drops under the four scenarios. With regard to LC scenario, the carbon emission intensity will be 1.31 *tC/10^4^yuan* by 2020, reduced by 46.36% on the level of base year. It is easy to realize the target that CO_2_ emissions will be reduced by nearly 40% in 2020 in China. But this reduction is reached at the cost of reduced economic growth. The growth rate of GDP per capita is below 6% under LC scenario and ELC scenario in China. In view of current social development in China, LC scenario is more feasible and practical than ELC scenario under the precondition that the target of carbon emission reduction established by Chinese government can be realized.

Besides China’s mitigation target of 40%–45% reduction of CO_2_ emission intensity by 2020, there is also a goal of increasing the share of non-fossil fuels in primary energy consumption to around 15% by 2020 compared with the 2005 level [7], the GDP growth rate of 6% was assumed according to economic development target until 2015 in China’s 12^th^ *Fiv*e-*Year Plan* [48]. With the gradual change of economic development mode in the planning, the GDP growth rate is supposed to further decline after 2015. So the 6% growth was also defined as a reference threshold of GDP growth rate in 2020 to judge the feasibility of scenarios.

## Conclusions and Path Selection

Judged according to actual situations of social and economic development in China, LC scenario is the most appropriate, the most feasible and the most practical scheme for low-carbon transformation in China and can maximize harmonious development of economy, society, energy and environmental systems. Therefore, under LC scenario, the paper, according to analysis results of scenario indexes, provides four paths of China’s low-carbon transformation to be selected: Technological innovation, industrial structure optimization, energy structure optimization and policy guidance. The low-carbon transformation process will be promoted through corresponding path selection in China in the future.

1. Technological innovation. Low carbon technology innovation contributes to the drop in energy intensity to restrict total energy consumption and growth rate of CO_2_ emissions and reduce carbon intensity. The carbon emission reduction technology is the driving force to improve energy utilization ratio; the clean utilization of high-carbon energy can be realized if advanced and efficient energy conservation and low carbon technology can be widely applied and relevant policies and measures can effectively promote carbon emission reduction and low-carbon development. China shall integrate technological research & development, policy orientation, economic development and climate change measures, make the best of existing natural resources, emphasize the development, conversion and utilization technology of major clean energy, realize low-carbon utilization of high-carbon energy and ensure that low carbon technology innovation is beneficial for sustainable development of energy and also for harmonious development of the economy. The technological innovation path is applicable to most regions and cities in China, and is especially influential in industrial and resource-based cities.
2. Industrial structure optimization. Based on natural resource endowment and other factors, the industry system with resource-based industry in dominance is formed in China. The energy consumption is huge in resource-based industry so that China’s industrial structure is featured with high energy consumption and high emission at present. Under LC scenario, Chinese government shall further optimize and adjust industrial structure, appropriately control high-energy consumption industry, positively take measures to promote low-carbon industries in development and gradually form the industrial structure with low energy consumption and high efficiency. The industrial structure optimization has a significant influence on China’s low-carbon development in the future.
3. Energy structure optimization. It is difficult to change the energy structure in which coal is dominant and which is formed upon resource endowment in China in a short period. The short-term “low carbon” adjustment of energy structure is beneficial for the reduction of CO_2_ emissions, but has little influence for low-carbon development, thus, the breakthrough in new energy technology is required. Apart from reasonable mining and processing of fixed energy, Chinese government shall attach importance to new energy and renewable energy to optimize energy structure. In the short term, coal consumption shall be continually reduced, while petroleum and natural gas shall be increased appropriately in consumption. In the long term, renewable resources such as wind energy, hydroenergy, bioenergy, solar energy and ocean energy shall be emphasized in the development. It is necessary to shrink the share of coal in energy consumption structure and gradually increase the proportion of new energy and renewable energy in consumption.
4. Policy guidance. Technical innovation, industrial structure optimization and energy structure optimization have a positive influence on China’s low-carbon development, but they must be guided by relevant policy. The government shall improve relevant energy conservation and emission reduction policies by formulating appropriate industrial structure upgrading strategies and adopting a motivation system of low carbon technology innovation in order to form an ideal low-carbon industry system characterized by low energy consumption, high technological content and good economic benefits, and to provide the positive policy orientation for low-carbon development in China. This path is the only way for China’s sustainable development in the future.
